# White Epidermoid Cyst: An Illustrative Case

**DOI:** 10.7759/cureus.75830

**Published:** 2024-12-16

**Authors:** Danielle C Brown, Rahul Garg, Mark Gedrich, Manisha Koneru, Daniel Tonetti

**Affiliations:** 1 Neurological Surgery, Cooper Medical School of Rowan University, Camden, USA; 2 Radiology, Cooper University Hospital, Camden, USA; 3 Neurological Surgery, Cooper University Hospital, Camden, USA

**Keywords:** epidermoid cyst, hyperdense epidermoid, intracranial, neuroradiology, white epidermoid cyst

## Abstract

White epidermoid cysts are a rare type of epidermoid cyst that appears hyperintense on T1 weighted magnetic resonance imaging (MRI) and are known for their characteristic pearly white outer appearance on gross pathology. White epidermoid cysts are not common findings; therefore, this illustrative case report was written to highlight the characteristics, progression, radiological evaluation, and management of a patient who presented to our center. This case report emphasizes the importance of determining the proper diagnosis to treat and manage the condition appropriately and avoid potential future complications.

## Introduction

Epidermoid cysts are thin-walled tumors that are benign and develop slowly [1]. They are often characterized as having a “pearly” outer appearance and are surrounded by stratified squamous epithelium [1,2]. They can be found in many locations, with the prevalence of intracranial epidermoid cysts being 0.3%-1.8% of intracranial tumors [3]. These cysts are of ectodermal origin and often arise due to failed detachment from the neural tube early in development. However, they are typically not identified until the third to fifth decade of life [4,5]. To identify epidermoid cysts, specific magnetic resonance imaging (MRI) and computed tomography (CT) scan characteristics can be utilized. On MRI, the epidermoid cyst is T1 hypointense and T2 hyperintense [6]. On CT, epidermoid cysts often appear hypodense [2]. If the radiological findings are contradictory to those mentioned above, a special type of epidermoid cyst can be identified, the “white epidermoid” cyst. These appear hyperintense on T1 MRI, most likely due to high protein content [6]. Epidermoid cysts are typically treated surgically, with early resection being the standard approach to alleviate accompanying symptoms [7]. Here we present an illustrative case of a patient with progressive neurologic decline and seizures, later diagnosed as a white epidermoid cyst, initially misdiagnosed as a hemorrhagic stroke.

## Case presentation

History and presentation

**Figure 1:**
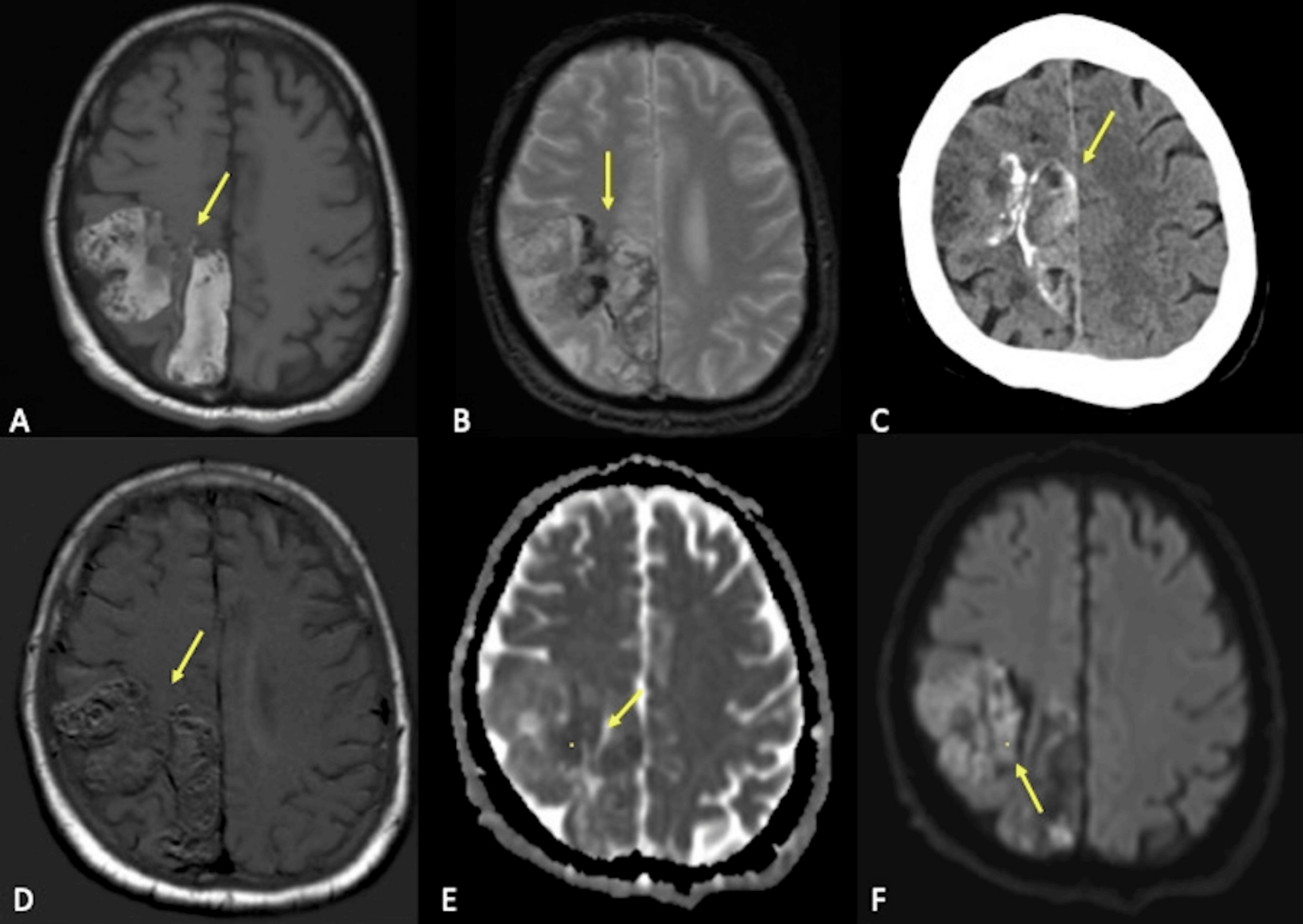
Axial CT and MRI sequences A: An axial T1-weighted sequence at the level of the centrum semiovale demonstrates a heterogeneous but predominantly T1 hyperintense lesion in the right frontoparietal region. B: An axial gradient echo sequence shows paramagnetic susceptibility within the lesion, correlating with calcification on CT. C: An axial non-contrast CT scan depicts the right frontoparietal lesion containing calcifications and internal areas of hyperattenuation, corresponding to T1 hyperintensity on MRI, suggestive of proteinaceous contents. D: An axial T1 post-contrast subtraction sequence shows a lack of enhancement in the right frontoparietal lesion. E and F: Axial diffusion and ADC show limited areas of restricted diffusion (yellow dot) in the right frontoparietal lesion. CT: computed tomography; MRI: magnetic resonance imaging; ADC: apparent diffusion coefficient

A 73-year-old male who was an avid cyclist presented to his primary physician with acute onset, painless left lower extremity weakness over the preceding week. CT imaging revealed a right frontoparietal hyperdense lesion, interpreted as subacute intraparenchymal hemorrhage (Figures 1A-1F). A detailed history revealed that the patient had a focal, uncontrolled left leg shaking episodes occurring when he was experiencing fatigue or after long cycling rides; he had never sought medical attention for these involuntary episodes. 

**Figure 2:**
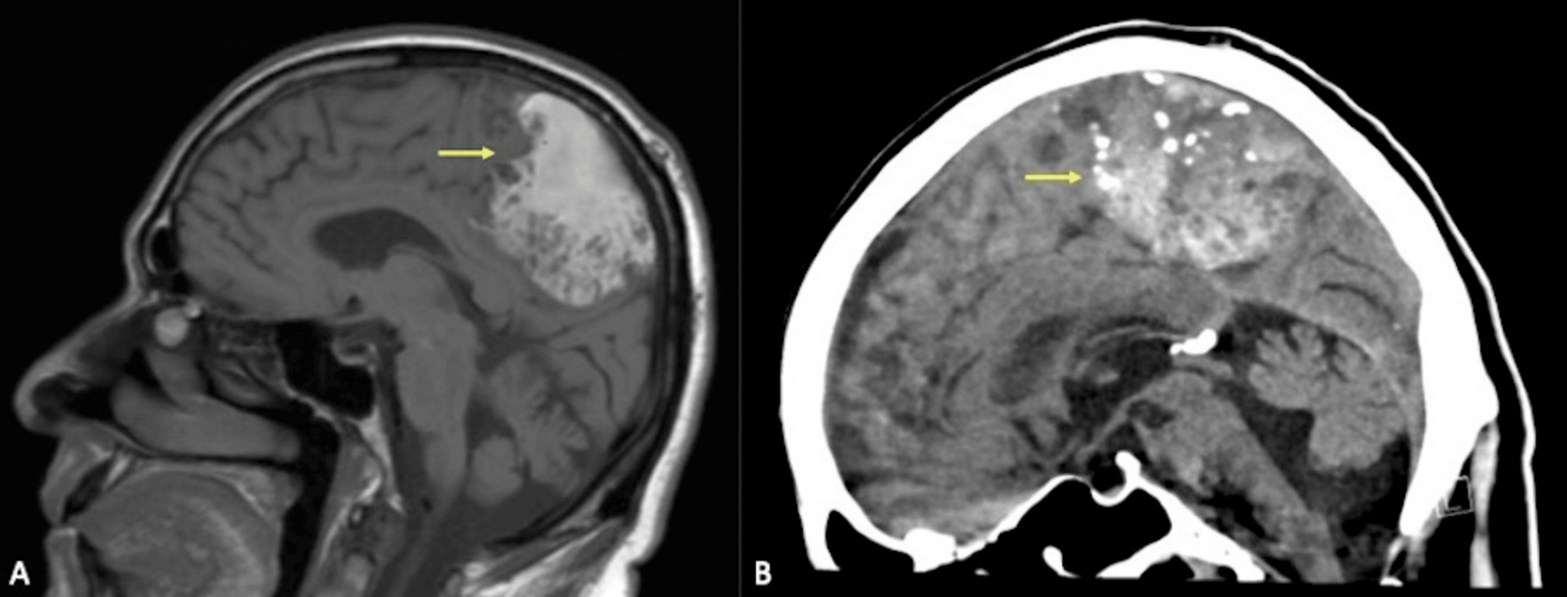
Sagittal CT and MRI sequences A: A sagittal non-contrast T1-weighted sequence depicts a markedly T1 hyperintense right frontoparietal lesion. B: A sagittal non-contrast CT scan again shows calcifications and hyperattenuation in the right frontoparietal lesion, corresponding to T1 hyperintensity on MRI and therefore indicative of intralesional high-protein content. CT: computed tomography; MRI: magnetic resonance imaging

**Figure 3:**
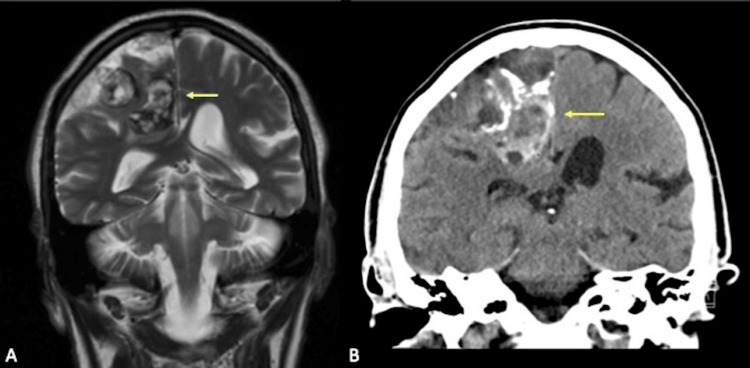
Coronal CT and MRI sequences A: Coronal T2-weighted sequence demonstrates a heterogeneous lesion in the right frontoparietal region with peripheral T2 hypointensity, correlating to calcification. B: Coronal CT shows hyperattenuation in the right frontoparietal lesion and calcification, correlating to T2 hypointensity on MRI. CT: computed tomography, MRI: magnetic resonance imaging

Two months after the onset of acute worsening left leg weakness, manifesting as painless weakness in hip flexion and knee extension, he was referred to our neurological institute after his symptoms failed to improve with physical therapy. Detailed neurologic examination revealed decreased proprioception, vibration, and left lower extremity weakness. Lumbar puncture analysis revealed elevated cerebrospinal protein levels but was otherwise normal and serum laboratory values were normal. MRI revealed a T1 hyperintense lesion of the right parasagittal frontoparietal region (Figures 1A-1F, 2A, 2B) with a heterogenous T2 signal throughout the lesion (Figures 3A, 3B). Scattered calcifications were found throughout and on the surface of the lesion (Figures 1A-1F). 

Diagnosis and outcomes

At the initial presentation, a wide range of differential diagnoses were considered. A toxic-metabolic cause, other spinal pathology, and malignancy were considered given recent unintentional weight loss and symptoms of fatigue and chills. Lack of symptoms and history suggestive of cauda equina syndrome ruled this out as a possibility. Guillain-Barre was considered but was ruled out due to no evidence of the pathognomonic ascending weakness. Infectious cause was ruled out due to the lack of fever. The most likely differentials included malignancy, such as low-grade glioma, or hemorrhagic transformation secondary to ischemic strokes.

While pathological analysis of a brain biopsy was utilized to identify the specimen as an epidermoid cyst, ultimately, imaging review and correlation of hyperintense signal on T1 MRI identified the cyst as a white epidermoid cyst.

## Discussion

Epidermoid cysts are benign lesions of ectodermal origin that are characterized by their stratified squamous exterior and “pearly” outer appearance [1,2,4]. The cyst’s components are often keratin produced by the lining of the cyst, degenerated blood components, and clefts of cholesterol [8]. They are typically asymptomatic; however, they may lead to inflammation and symptoms upon rupture of the cyst [8]. Epidermoid cysts are identified on MRI by their hypointense signal on T1-weighted imaging, the hyperintense signal on T2-weighted imaging, profound hyperintensity on diffusion-weighted imaging, and associated hypointensity on the apparent diffusion coefficient (ADC) map. They additionally present with hypodensity on CT, resembling cerebrospinal fluid (CSF) [6]. Intracranially, they are typically located off-midline or laterally [6]. 

White epidermoid cysts have some notable structural differences in comparison to classical epidermoid cysts and thus present differently on MRI and CT. White epidermoid cysts have triglycerides that the classical epidermoid cysts lack, along with a more elevated protein content [9]. White epidermoid cysts are identified mainly by their hyperintense T1 signal, along with a hypointense T2 signal, and hyperdensity on CT [6]. Portions of the lesion may also restrict diffusion. Approximately 3% of all epidermoid cysts are labeled as white epidermoid cysts [10]. The recognition and inclusion of white epidermoids in the differential diagnosis of hyperdense lesions on CT is important, as they can cause symptoms following rupture and are at considerable risk for the development of aseptic meningitis [9]. 

In this present case, a white epidermoid cyst masqueraded as a subacute intraparenchymal hemorrhage based on the imaging appearance on CT and MRI scans. Because this subacute course seemed to match the patient’s history, this seemed an appropriate initial diagnosis. However, a tissue biopsy and then ultimately serial imaging studies revealed the presence of a white epidermoid cyst.

The neuroimaging demonstrated a lesion with internal marked hyperintense signal on T1-weighted imaging with corresponding hyperattenuation on CT indicative of high protein content, and scattered calcifications on both CT and MRI. This appearance can be contrasted with the typical epidermoid cyst, which demonstrates a hypointense T1 signal, hyperintense T2 signal, restricted diffusion throughout, and CSF attenuation on CT. Taken altogether, the diagnosis of a white epidermoid cyst should be considered by practitioners caring for patients with atypical appearing intracranial lesions with marked T1 hyperintensity on MRI, as the definitive treatment of complete surgical resection relies on accurate radiographic diagnosis.

## Conclusions

Intracranial epidermoid cysts presenting with marked T1 hyperintense signal on MRI are classified as white epidermoid cysts and are rare central nervous system neoplasms. Early diagnosis of these cysts is important to prevent symptoms associated with cyst rupture. This case demonstrates a rare presentation of a supratentorial white epidermoid cyst. These lesions can initially be misdiagnosed as other pathologies with similar presentations and radiographic appearances, which emphasizes the importance of accurate radiographic analysis and clinical suspicion in aiding definitive treatment.
